# ESTERR-PRO: A Setup Verification Software System Using Electronic Portal Imaging

**DOI:** 10.1155/2007/61523

**Published:** 2006-11-29

**Authors:** Pantelis A. Asvestas, Konstantinos K. Delibasis, Nikolaos A. Mouravliansky, George K. Matsopoulos

**Affiliations:** Institute of Communication and Computer Systems, National Technical University of Athens, 9 Iroon Polytechniou Street, Zografos, Athens 15780, Greece

## Abstract

The purpose of the paper is to present and evaluate the performance of a new software-based registration system for patient setup verification, during radiotherapy, using electronic portal images. The estimation of setup errors, using the proposed system, can be accomplished by means of two alternate registration methods. (a) The portal image of the current fraction of the treatment is registered directly with the reference image (digitally reconstructed radiograph (DRR) or simulator image) using a modified manual technique. (b) The portal image of the current fraction of the treatment is registered with the portal image of the first fraction of the treatment (reference portal image) by applying a nearly automated technique based on self-organizing maps, whereas the reference portal has already been registered with a DRR or a simulator image. The proposed system was tested on phantom data and on data from six patients. The root mean square error (RMSE) of the setup estimates was 0.8 ± 0.3 (mean value ± standard deviation) for the phantom data and 0.3 ± 0.3 for the patient data, respectively, by applying the two methodologies. Furthermore, statistical analysis by means of the Wilcoxon nonparametric signed test showed that the results that were obtained by the two methods did not differ significantly (*P* value > 0.05).

## 1. INTRODUCTION

The effectiveness of radiation therapy depends on the patient setup accuracy at each radiation treatment session. A significant problem is to reproduce the intended position of the part of the patient that is irradiated with respect to the treatment beam(s) at each treatment session. It is a common clinical practice to verify the setup by comparing the portal image with a reference one which records the intended patient position. Typical reference images that can be used are simulator images, digitally reconstructed radiographs (DRRs), or another portal images. The introduction of electronic portal imaging devices (EPIDs) offers the potential for correcting inaccuracies in patient placement in a prospective manner, rather retrospectively as is done with conventional megavoltage films.

In general, setup errors are classified as random (or interfraction) and systematic errors [1]. The random errors are deviations between different fractions, during a treatment series, whereas the systematic errors are deviations between the intended patient position and the average patient position over a course of fractionated therapy [2]. Furthermore, random patient movement or periodic movements such as breathing can cause the so-called intrafraction error, which is defined as the deviation observed within a single fraction of fractionated therapy. However, these movements during a single fraction are usually insignificant for most patients and treatment sites, with a few exceptions (e.g., the lung).

A number of setup correction strategies aiming at improving target localization during radiation therapy treatments have been proposed. Most of these strategies are based on the matching of common anatomical features of portal images selected either manually or semiautomatically. In [3], the magnitude of the errors introduced into the registration between the rotated and the nonrotated phantom images and the reference DRR image was determined based on “match structures,” which include the field edges and at least three anatomical landmarks manually selected on the reference image and matched with the corresponding anatomy in the portal images. An object-based registration method for portal images was developed in [4], which was based on core analysis, a fundamental computer vision method, to define correspondence between common anatomical structures of the images manually selected and a curved-based matching algorithm (chamfer-matching) in order to determine the translation/rotation parameters of the image registration. Similar approaches for registering DRRs with portal images were also proposed using either the Pearson's correlation coefficient as a measure of match on selected anatomical features [5] or the template matching technique [6]. Other strategies for the verification of patient setup were based on the optimization of a similarity measure such as histogram matching technique [7], phase-only correlation [8], the minmax entropy [9], and the mutual information [10]. Furthermore, a comparative study of various similarity measures and optimization procedures had been performed on matching high-quality DRRs against portal images that were acquired right before treatment dose delivery [11].

Setup errors that are caused by out-of plane rotations can be estimated by means of three-dimensional techniques [5, 12]. In general, out-of-plane rotational errors that are smaller than 3° do not affect the projected anatomy in portal images significantly. However, when larger rotational errors are not taken into account, this causes a reduced accuracy in the measurement of the translational error [13]. In some cases, two-dimensional techniques can also provide reliable estimations of the out-of-plane rotational errors; out-of-plane rotation for an anterior image can be an in-plane rotation for a lateral image.

In this paper, an extended version of our paper in [14] is presented for the estimation of patient setup errors during radiotherapy treatments. According to the proposed methodology, the verification of patient setup consists of the following steps: (a) delineation of radiation field edges in the portal image in order to verify that the beam has the correct shape as well as to establish a common coordinate system with a previously delineated field edge from the reference image, and (b) matching of common anatomical structures within the two images in order to provide an estimation of the patient setup error relative to the field edges. Two registration methodologies are presented within the paper: (a) the registration of portal image of the current fraction of the treatment with the corresponding DRR image, used as a reference image and (b) the registration of portal images at different treatment sessions using a nearly automatic technique based on self-organizing maps (SOMs) to define automatic correspondence of common anatomical features of the portal images. Both registration methodologies have been incorporated towards the development of a software system, called ESTERR-PRO, for the estimation of patient setup errors as presented in the paper. A detailed description of the system in terms of registration methodologies is provided in Section 2. In Section 3, results of the performance of the system on phantom and real data are presented. Finally, in Sections 4 and 5, discussion of the results and concluding remarks are drawn, respectively.

## 2. MATERIALS AND METHODS

### 2.1. The proposed software system: “ESTERR-PRO”

The complete system incorporates the following features: (a) friendly user interface, (b) image processing tools, and (c) a database of patient records and images. The user of the system is able to access and display portal images and corresponding reference images (DRRs simulator images) for a patient selected from the database. The image processing tools can then be used to detect and objectively estimate the setup error, if present.

ESTERR-PRO runs on personal computers (PCs) under the Microsoft Windows operating system. The software was developed in the C++ language.

**Figure 1:**
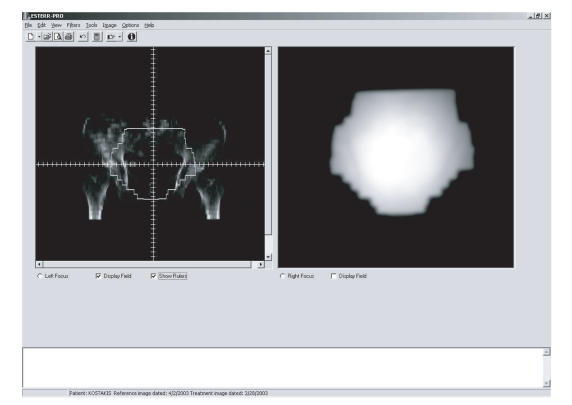
The ESTERR-PRO interface. The main screen is divided into two side-by-side panels. A portal image is displayed on the right panel and a reference image is displayed on the left panel.

In order to review portal images, the user must first select a patient name from the database. Then all the available reference and portal images, sorted by date, are presented to the user. When the selection of the reference and the portal image is completed, the images are displayed next to each other (see Figure 1).

The image processing toolkit includes tools for (a) preprocessing such as brightness/contrast adjustment, contrast enhancement (histogram equalization [15], contrast and limited adaptive histogram equalization [16]), and smoothing (mean filtering, median filtering, morphological smoothing [15]), (b) patient setup verification.

### 2.2. Patient setup verification

As already mentioned, the patient setup verification comprises two steps: (a) delineation of radiation field edges in the portal image in order to verify that the beam has the correct shape as well as to establish a common coordinate system with a previously delineated field edge from the reference image, and (b) matching of common anatomical structures within the two images.

#### 2.2.1. Verification of field shape

The radiation field edges in the portal image are delineated automatically as follows: a thresholding operation (with threshold level set to 5) of the gray-levels of the image is applied in order to obtain a rough approximation of the field contour. Then, the Canny edge detector [17] using a fast recursive implementation of the Gaussian kernel [18], applied on the original image in a band of width 15 pixels around the position of the initial contour, provides the final form of the field edges. The values for the standard deviation of the Gaussian kernel, the low threshold, and the high threshold for the nonmaximum suppression of the Canny edge detector were set to 2.0, 0.0, and 0.95, respectively. The field edges for each image are stored in the computer memory as a binary image, which is called *field edge map* and has the same size as the original image. A value 1 (0) in the field edge map indicates that the corresponding pixel belongs to the field contour (background).

The verification of the field shape is accomplished automatically as follows: first, the distance transformation of the reference field edge map is calculated [19]. The distance transformation provides the smallest distance of each pixel of the field edge map from the field edge. Then, an optimization process is applied in order to achieve the spatial coincidence of the two radiation field edge maps. The optimization process involves the minimization, with respect to the parameters of a rigid transform (namely displacement and rotation in the image plane) of the distance between the reference edge map and the transformed version of the edge map of the portal image, using the current values of the parameters of the rigid transform. The distance between the two edge maps is calculated by means of the distance transformation of the reference field edge map. In our implementation, the Powell's method [20] is used for the optimization process. After the end of the optimization process, the distance between the reference edge map and the transformed edge map of the portal image should be small enough. If this distance exceeds a predefined value, this means that the two field edges do not have the same shape and a warning is generated, which informs the user about this inconsistency. Additionally, if the reference image is another portal image then it is expected that the parameters of the rigid transform, obtained during the optimization process, to be nearly zero. If this is not the case, then a warning is also generated. It must be noted that the whole process is invoked automatically, immediately after the user selects the pair of the images.

#### 2.2.2. Matching of anatomical structures

The choice of the procedure that is used for the matching of common anatomical structures between the reference and the portal image depends on the type of the reference image: (a) if the reference image is a DRR or a simulator image, a modified manual procedure is applied, (b) if the reference image is a portal image, a semiautomatic approach is followed.

**Figure 2:**
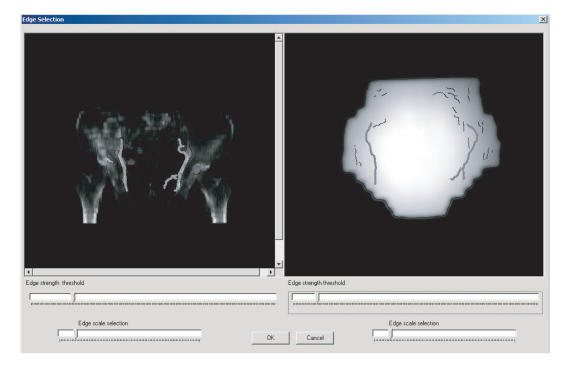
The edge selection window for matching the anatomical structures between a DRR and a portal image.

In particular, when the reference image is a DRR or a simulator image, the following procedure is applied. The edges of each image are extracted automatically by the Canny edge detector. Trackbars for the adjustment of the values of the parameters Canny edge detector (namely, the standard deviation of the Gaussian kernel and the high threshold for the nonmaximum suppression) are available (see Figure 2) in order to provide the capability of the user to select only edges that correspond to the anatomical structures of interest.

When the user selects the edges that correspond to the same anatomical structures within the two images, a similar optimization procedure as the one used for the verification of the field shape is invoked. The outcome of the aforementioned procedure is the setup error in terms of horizontal displacement, vertical displacement, and angle of rotation around the axis that is perpendicular on the image plane.

When the reference image is another portal image, the matching procedure involves the definition of fiducial marks (points) on anatomical structures of the reference image that do not move with time, or with anatomical processes (the skeletal system is a good example, whereas the bladder or the intestines are counterexamples of placement of fiducial marks). The user-defined fiducial marks can be saved with the reference image to be utilized in subsequent fractions of the radiation treatment series. Let (*x_i_*, *y_i_*) (*i* = 1, 2,…, *N*) be the coordinates (in pixel units) of the user-defined fiducial marks. The next step of the procedure involves the maximization of a properly chosen function, *f*, with respect to the parameters of the setup error (horizontal displacement (*dx*), vertical displacement (*dy*), and angle of rotation (*θ*)) using self-organizing maps [21]. The self-organizing map (SOM) is a neural network algorithm, which uses a competitive learning technique to train itself in an unsupervised manner. Kohonen first established the relevant theory and explored possible applications [22]. The Kohonen model comprises a layer of neurons *m*, ordered usually in a one- or two-dimensional grid. The training of the network is performed in an iterative way. At each iteration *k*, a data point **x** ∈ ℝ^*n*^ is presented to the network; the neuron *j* with weight vector **w**_*j*_ ∈ ℝ^*n*^ is declared as the winning neuron, according to the following rule:

(1) $$j=\underset{i}{\text{arg min}}\left(\left\|\text{x}-{\text{w}}_{i}\right\|\right).$$

The winning neuron *j* and its neighboring neurons *i* have their weight vectors modified according to the following rule:

(2) **w**_*i*_(*n* + 1) = **w***_i_*(*n*) + *h_ij_*(*n*)
[**x**(*n*) − **w***_i_*(*n*)],

where *h_ij_*(*n*) = *h*(‖**r** *_i_* − **r** *_j_*‖, *n*) is a kernel defined on the neural network space as a function of the distance ‖**r** *_i_* − **r** *_j_*‖ between the winning neuron *j* and its neighboring neurons *i*, as well as the iteration number *n*. This kernel has the shape of the “Mexican hat” function, which in its discrete form has maximum value at inter-neuron distance in the case of *i* = *j* whereas its value drops in a Gaussian manner as the distance increases. The width of this function decreases monotonically with iteration number. In this way convergence to the global optimum is attempted during the early phases of the self-training process, whereas gradually the convergence becomes more local as the size of the kernel decreases.

Prior the description of the proposed method, some notations must be introduced. Let *μ_A_* (*I*) denote the restriction of an image *I* to the region *A* ⊂ ℝ^2^ and *T*_**w**_(*A*) ⊂ ℝ^2^ is the rigid transformation, with parameters **w** = (*dx*, *dy*, *θ*), of the region *A*, where *dx*, *dy*, and *θ* are the horizontal displacement, the vertical displacement, and the angle of rotation, respectively. Furthermore, MoM(*I*_1_, *I*_2_) denotes a measure of match between two images *I*_1_ and *I*_2_.

If *I_R_*, *I_F_* are the reference image and the image to be registered, respectively, then the implementation of the SOM network for registering the two images is as follows. The topology of the network is constructed by placing a neuron on each user-defined fiducial mark **P** *_i_* = (*x_i_*, *y_i_*) (*i* = 1, 2,…, *N*) of the reference image. Each neuron is associated with a square area *A_i_* = [*x_i_* − *R*, *x_i_* + *R*] × [*y_i_* − *R*, *y_i_* + *R*], of (2*R* + 1)^2^ pixels, centered at the position of the neuron. Additionally, a weight vector **w** *_i_* = (*dx_i_*, *dy_i_*, *θ _i_*), which holds the parameters of a local rigid transformation, is assigned to each neuron.

The SOM network is trained as follows.

1. For each neuron, the components of the weight vector are initialized to zero values, **w** *_i_* (0) = (0, 0, 0), the quantities MoM*_i_*(0) ≡ MoM(*μ_A_i__* (*I_R_*), $\mu_{T_{w_{i\left(0\right)}}\left(A_{i}\right)}\left(I_{F}\right)$) are calculated, the variable MoM_best_ is set to a very large (in magnitude) negative value, and the iteration variable, *n*, is set to 1.
2. While *n* is less than *n*_max_,
  - if the average value of the MoM*_i_* (*n* − 1), MoM_ave_(*n* − 1), is better than MoM_best_, then MoM_best_ = MoM_ave_(*n* − 1) and the current weights are stored as **w** *_i_*;
  - an *input signal*, **s**(*n*) = (*dx*(*n*), *dy*(*n*), *θ*(*n*)), is generated randomly;
  - for every neuron, the quantity MoM*_i_*(*n*) ≡ MoM(μ*_A_i__*(*I_R_*),μ_T_**s**(*n*)__(*A_i_*)(*I_F_*)) is calculated;
  - the *winning neuron*, *k_n_*, in the current iteration, is defined as
    (3) $$k_{n}=\underset{i}{\text{arg min}}\left\{{\text{MoM}}_{i}\left(n\right)\right\}$$
    under the condition
    (4) MoM*_k_n__* (*n*) > MoM_ave_(*n* − 1);
  - the weights of the neurons are updated according to the following equation:
    (5) **w***_i_*(*n*) = **w***_i_* (*n* − 1) + *h*(*k_n_*, *n*, *i*) [**s**(*n*) − **w***_i_* (*n* − 1)],
    where *h*(*k_n_*, *n*, *i*) (*i* = 1, 2,…,*N*) is given by the following equation:
    (6) $$\begin{array}{ll} h\left(k_{n},n,i\right) & =\{\begin{array}{ll} L^{q^{\left(n\right)}}, & \left\|{\text{P}}_{k_{n}}-{\text{P}}_{i}\right\|<\alpha^{q^{\left(n\right)}}d_{0},\\ 0 & \text{otherwise} \end{array}\\ & q(n)=\left\lfloor \frac{n}{p+1}\right\rfloor , \end{array}$$
*L*, *a*, *d*_0_ ∈ ℝ and *p* ∈ ℝ are parameters to be defined later, ‖‖ denotes the Euclidean norm, and ⌊ ⌋ is the floor function;
  - the iteration variable is increased by one.
3. When the training is finished, the parameters of the affine transformation between the two portal images are calculated using a least squares method between the point sets {**P** *_i_*} and {*T*_**w**_*i*__(**P** *_i_*)} [20].

The selected measure of match was the gradient correlation coefficient, namely,

(7) $$\begin{array}{ll} \text{M}\text{o}\text{M}(I_{R},I_{F}) & =GCC\left(I_{R},I_{F}\right)\\ & =GCC_{h}(I_{R},I_{F})+GCC_{v}(I_{R},I_{F}), \end{array}$$

where

(8) $$\begin{array}{c} GCC_{h}\left(I_{R},I_{F}\right)=\frac{{\left\{\sum_{x,y}\left[G_{R}^{h}\left(x,y\right)-{\bar{G}}_{R}^{h}\right]\left[G_{F}^{h}\left(x,y\right)-{\bar{G}}_{F}^{h}\right]\right\}}^{2}}{\sum_{x,y}{\left[G_{R}^{h}\left(x,y\right)-{\bar{G}}_{R}^{h}\right]}^{2}\sum_{x,y}{\left[G_{F}^{h}\left(x,y\right)-{\bar{G}}_{F}^{h}\right]}^{2}},\\ GCC_{v}\left(I_{R},I_{F}\right)=\frac{{\left\{\sum_{x,y}\left[G_{R}^{v}\left(x,y\right)-{\bar{G}}_{R}^{v}\right]\left[G_{F}^{v}\left(x,y\right)-{\bar{G}}_{F}^{v}\right]\right\}}^{2}}{\sum_{x,y}{\left[G_{R}^{v}\left(x,y\right)-{\bar{G}}_{R}^{v}\right]}^{2}\sum_{x,y}{\left[G_{F}^{v}\left(x,y\right)-{\bar{G}}_{F}^{v}\right]}^{2}}. \end{array}$$

The subscript *R*(*F*) refers to the reference (to be registered) image; the superscript *h*(*v*) refers to the horizontal (vertical) direction in the image plane, and *G* denotes the first derivative. For example, *G_R_^h^*(*x*, *y*) denotes the first derivative of the reference image along the horizontal direction estimated at pixel position (*x*, *y*). $\bar{G}$ refers to the mean value of the first derivative.

The rationale for selecting the aforementioned measure of match was that gradient measures concentrate the contributions on edge information, which intuitively appears sensible.

The following generator of random numbers is used for producing the input signals to the network:

(9) $$\begin{array}{ll} s_{j}\left(n\right) & =w_{k_{n},j}+\text{sgn}\left(v_{i}-0.5\right)\text{TM}\left(n\right)\\ & \;\times [{\left(1+\frac{1}{\text{TM}\left(n\right)}\right)}^{\left|2v_{j}-1\right|}-1]\left(U_{j}-L_{j}\right)\left(j=1,2,3\right)\\ \text{T}\text{M}\left(n\right) & =\{\begin{array}{ll} 1, & n=0,\\ \text{exp}\left(-2{\left(q\left(n\right)\right)}^{1/n}\right), & n>0, \end{array} \end{array}$$

where *s*_1_ (*n*) = *dx*(*n*), *s*_2_ (*n*) = *dy*(*n*), *s*_3_ (*n*) = *θ* (*n*), *v_j_* is a uniformly distributed random variable in [0, 1], and *U_j_*(*L_j_*) denotes the maximum (minimum) allowed value for the corresponding component of the input signal. Although *U_j_* and *L_j_* are inputs to the matching process, for all pairs of images used in the current study, constant values were used (±50 pixels for the displacement and ±10° for the angle of rotation).

**Figure 3:**
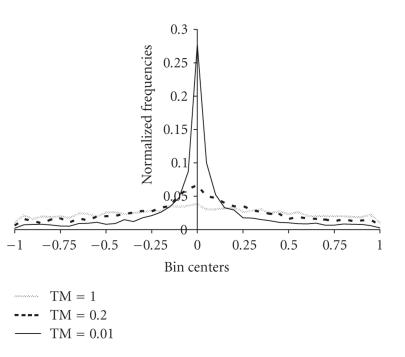
Normalized histogram of the values obtained by means of the random number generator described in (9) for different values of the parameter *TM*.

It must be noted that (9) is a slightly modified version of the generator used in the very fast simulated annealing method [23] and provides random signals which in general lie in the range [$w_{k_{n},j}$ − (*U_j_* − *L_j_*), $w_{k_{n},j}$ + (*U_j_* − *L_j_*)]. When a generated signal is not in the allowed range [*L_j_*, *U_j_*], then it is discarded and a new signal is produced until *s_j_*(*n*) ∈ [*L_j_*, *U_j_*]. The parameter TM(*n*) controls how far from the weights of the current winning neuron the input signal can reach. As the iteration variable evolves, the magnitude of TM(*n*) falls exponentially and the generated input signals are more localized around the weights of the current winning neuron (see Figure 3). This is a desired property, since as the number of iterations grows, the weights of the current winning neuron get closer to the parameters of the solution of the matching problem.

The parameter *d*_0_ provides the initial radius of a circular region around the winning neuron. Only neurons inside this region are updated. Usually, *d*_0_ is set to the maximum distance between the fiducial marks. As can be seen from (6), this distance is reduced with geometric rate determined by the parameter *α* (0 < *α* ≤ 1). A typical value for the parameter *α* is 0.995. The parameter *L* acts like a gain constant for the magnitude of the update that is applied to the weights of the neurons. This parameter also decreases geometrically as the iteration variable evolves. The range of values *L* is between 0.99 and 1.0; a typical value is 0.995. The parameter *p* is an integer that determines the rate of change of the parameters *L* and *α*. Practically, this parameter determines the number of iterations that are executed before an adjustment of the values for the parameters *L*, *α*, and TM(*n*) takes place. A typical value for this parameter is 200. The number of iterations is set to 5000 and the size of the square area associated with each neuron is 19 (*R* = 9).

Finally, since the transformed region *T_**s**_*(*n*)(*A_i_*) does not have integer coordinates, bilinear interpolation is used in order to calculate MoM*_i_*(*n*).

**Figure 4:**
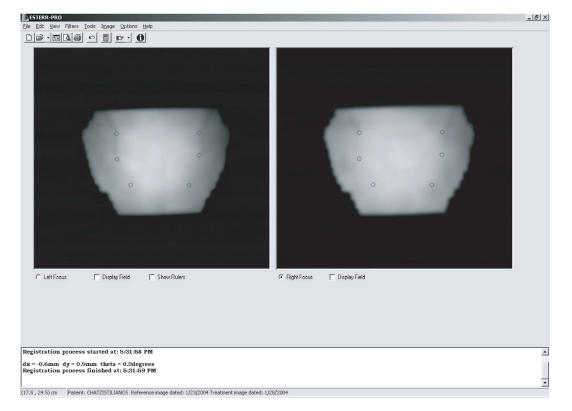
Example of matching a pair of portal images on patient data. The fiducial marks, defined by the user, are shown on the reference image (left panel). The corresponding points on the other portal image, after the end of matching procedure, are also shown.

In Figure 4, the results that were obtained from the application of the proposed methodology on real pelvic data are presented.

### 2.3. Data acquisition and evaluation protocol

The proposed system was tested on phantom and real data. All the portal images were acquired for gantry angle 0° using a CCD camera-based EPID (Beamview Plus v2.1, Siemens) of “HYGEIA” with a total dose of ∼ 20 MU/image; 6-MV X-rays were used. The size of the pelvic field was 12 × 12 cm^2^ at the isocenter. The DRR images were obtained from a CT data set acquired using a Siemens Somaton Plus 4 scanner at 120 keV, with a 2 mm slice thickness and no gap between slices.

An anthropomorphic phantom (Alderson Rando phantom), commercially available system, was used. The evaluation method was as follows.

- A DRR and a portal image of the selected region of interest (head, lung, pelvis) of the phantom, with no setup error between them, were acquired. This was achieved by means of three fiducial markers, visible on the CT imaging, adhered on the phantom using laser alignment to define a reference point on both CT scanning and irradiation. This point was used as the isocenter during treatment planning (DRR) and irradiation (portal imaging). These DRR and portal images served as the reference images. Before the acquisition of CT phantom images and phantom irradiation, the accuracy of the lasers alignment in both the CT and the treatment room was checked and found within 1 mm. Moreover, possible introduced inaccuracies due to no-horizontal CT-couch motion or inaccuracies in the stated slice thickness must be excluded since an extensive quality control was performed prior to the use of the CT scanner.
- An expert from the “HYGEIA” hospital defined the fiducial marks on anatomical structures, (*x_i_*, *y_i_*) (*i* = 1, 2,…, *N*), on the portal reference image. These points were used for matching each portal image with the reference portal image.
- The treatment couch was moved along the horizontal (left-right) and/or vertical (head-foot) direction 2 mm, 4 mm,…, 12 mm and a new portal image was acquired. Before phantom irradiation, the treatment couch (ZXT-Siemens) reading positions were checked and their accuracy was found within 1 mm.
- The setup verification tool of the system was invoked in order to estimate the setup error between the reference images and each new portal image, using the procedures described in Sections 2.2.1 and 2.2.2. The output of the system was the estimated values of the parameters of the simulated setup error, namely horizontal displacement (mm), vertical displacement (mm), and angle of rotation (degrees).
- A set of 50 test points, **P** *_i_* (*i* = 1, 2,…, 50), was defined on the reference DRR. Since the setup error is known, the actual position of these points on the portal images (including the reference portal image) was identified. The position of these points on the portal images was also identified using the estimated values for the setup error. For each portal image, the root mean square error (RMSE) (in millimeters) between the actual and the estimated positions of this set points was calculated by the following equation:
  (10) $$\text{R}\text{M}\text{S}\text{E}=\sqrt{\frac{1}{50}\sum_{i=1}^{50}{\;\|\;{\left(P_{i}\right)}_{\text{a}\text{c}\text{t}}-{\left(P_{i}\right)}_{\text{e}\text{s}\text{t}}\;\|}^{2},}$$
  where the subscript act (est) refers to the actual (estimated) positions of the test points on the portal images and ‖ ⋅ ‖ denotes the Euclidean distance.

For the real data, the aforementioned evaluation procedure was slightly modified, since it is not known the actual setup error. Therefore, a manual registration, to serve as ground truth, was carried out by two experts from “HYGEIA” hospital. The average value of the two obtained estimates was used as ground truth. A total of six subjects were investigated, who were recruited from patients referred to the “HYGEIA” hospital for prostate cancer treatment. For each patient, a DRR and thirty portal images were acquired correspondingly. The proposed evaluation protocol has been approved by the ethical committee of the “HYGEIA” hospital and the subjects gave informed consent to the work.

### 2.4. Statistics

The statistical difference between the actual and estimated positions was assessed by means of the Wilcoxon signed nonparametric test, for both real and phantom data [24]. The null hypothesis was that the two methods (DRR—portal and portal—portal matching) did not differ as per the RMSE.

## 3. RESULTS

### 3.1. Phantom data

Setup error estimations (three parameters and RMSE) are shown in Tables 1 and 2, respectively. For the pelvic region, the RMSE was 0.8 ± 0.3 (mean value ± standard deviation) and 0.8 ± 0.4 when the reference image was the DRR and the portal image, respectively. For the cranial region, the RMSE was 0.8 ± 0.3 and 0.6 ± 0.3 when the reference image was the DRR and the portal, respectively. For both cases, the Wilxocon signed test showed that the null hypothesis could not be rejected at the 5% level (*P* value > 0.05).

### 3.2. Real data

For each patient, the average values of the setup error over the thirty portal images are shown in Table 3. The RMSE over all six patients was 0.3 ± 0.3 and 0.3 ± 0.3 when the reference image was the DRR and the portal corresponding to the first fraction of the treatment, respectively. The statistical analysis of the RMSE measurements of each patient showed that the null hypothesis could not be rejected at the 5% level (*P* value > 0.05).

## 4. DISCUSSION

The design and the development of the proposed system were based on several constraints. It should use the equipment available in the “HYGEIA” hospital: a portal imager and a CT scanner. The human intervention should be minimal, the results should be accurate and the execution time should be kept as low as possible.

In this framework, the estimation of the patient setup can be accomplished using two alternate processes: (a) the portal image corresponding to the current fraction of the treatment is matched directly with the DRR (or the simulator image). (b) The portal image is matched with the portal image acquired during the first fraction of the treatment (reference portal), whereas the reference portal has already been matched with the DRR image.

The rationale underlying the proposed system design was based on the following facts. In general, it is a very difficult task to achieve an accurate matching between a DRR and a portal image automatically, mainly due to the fact that the two images are acquired at totally different energies. It has been proposed to convert the DRR into a megavoltage DRR prior to the matching [5, 25, 26]. However, this was not possible in our case, due to software system installation into a dedicated computer platform, output incompatibility of the radiation treatment planning software and local network topology.

On the other hand, automated techniques based on segmentation should be excluded from the design since these methods rely heavily on the success of the segmentation step, which is a very difficult task due to the low inherent contrast of the portal images. Additionally, no segmentation technique can give satisfactory results for every anatomical region of interest. The very difficult task of portal image segmentation justified the development of other methods of research, such as intensity-based methods [5, 9, 27]. These methods assume there is a statistical relation between the gray level values of the pixels of the images to be matched and that this relation is at maximum when the images are matched. Although these methods seem to be promising, further work is required.

Another solution was the use of some kind of manual technique. However, pure manual methods depend on the accurate determination of homologous fiducial marks between the two images and furthermore are in general time consuming and prone to spatial inaccuracies. Therefore, regarding the match of DRR and a portal image, we have chosen a modified manual technique that automatically identifies candidate pairs of corresponding edges between the two images. Then, the user simply selects the proper pairs of edges that are going to be used for the matching. The results in Tables 1–3 indicate that the proposed methodology provides estimates of the setup error that are close enough to the expected ones. As can be observed, the values of patient displacements along the horizontal and vertical axis are smaller than those of the phantom, due to quality control processes adopted at the Radiotherapy Department of the “HYGEIA” Hospital.

As already mentioned, the estimation of the setup error can be also accomplished by means of a portal-to-portal matching method. This method requires the definition of a small number (four to seven) of fiducial marks only on the reference portal image. These fiducial marks are stored in the database and are automatically retrieved every time the specific patient is selected. The accuracy of the portal-to-portal matching lies within the limits imposed by the clinical routine. This approach introduces an additional error in the estimation of patient setup error (error due to the matching of DRR with the portal image of first fraction and error due to the matching of the portal of the current fraction with the portal image of the first fraction). However, statistical analysis showed that the two methods did not differ significantly as per the RMSE. Additionally, since a nearly automated method is used, the user is provided in return with a fast, reliable, robust, and user-friendly technique, which requires minimal user intervention.

## 5. CONCLUSIONS

An integrated software system has been presented for the calculation of patient setup errors in radiotherapy, using EPID images. The system handles both DRR-portal image pair as well as portal-portal pairs. The philosophy of the system was to achieve very fast execution time, increased robustness, considering parameter range as well as anatomic regions of the body and accuracy in the calculation of setup errors. The system has already been installed in the Radiotherapy Department of “HYGEIA” Hospital, Athens, and is fully operational in a clinical environment. The selected methods for image registration require minimal user intervention, achieve high accuracy, and have proven highly practical and popular among the physicians and physicists of the “HYGEIA” Hospital.

## Figures and Tables

**Table 1:** Setup error estimations (horizontal displacement, vertical displacement, angle of rotation, and RMS error) for known setup error of the pelvic region of the phantom for DRR versus portal and portal versus portal.

Expected			DRR versus portal				Portal versus portal			

*dx*(mm)	*dy*(mm)	*θ*(°)	*dx*(mm)	*dy*(mm)	*θ*(°)	RMSE(mm)	*dx*(mm)	*dy*(mm)	*θ*(°)	RMSE(mm)

2	0	0	3.0	0.1	−0.4	1.2	3.1	0.2	0.1	1.2
4	0	0	4.4	−0.1	0.2	0.6	4.8	−0.2	−0.1	0.8
6	0	0	5.5	−0.2	0.4	0.9	5.6	−0.2	−0.2	0.7
8	0	0	8.6	−0.8	0.3	1.2	9.3	−0.6	0.3	1.6
10	0	0	10.9	−0.7	0.3	1.3	10.1	−0.4	0.4	0.9
12	0	0	12.2	−0.3	0.0	0.4	12.2	−0.8	0.2	0.9
0	2	0	0.0	1.7	−0.2	0.4	0.1	1.4	0.1	0.6
0	4	0	−0.3	5.2	0.2	1.2	−0.1	4.9	0.1	0.9
0	6	0	−0.2	6.2	0.2	0.5	−0.1	5.6	−0.1	0.5
0	8	0	0.0	8.2	0.4	0.8	0.1	8.2	−0.1	0.2
0	10	0	0.5	9.5	0.0	0.8	0.1	9.6	0.3	0.7
0	12	0	−0.1	12.5	−0.1	0.6	−0.3	11.9	0.1	0.3

**Table 2:** Setup error estimations (horizontal displacement, vertical displacement, angle of rotation, and RMS error) for known setup error of the cranial region of the phantom for DRR versus portal and portal versus portal.

Expected			DRR versus portal				Portal versus portal			

*dx*(mm)	*dy*(mm)	*θ*(°)	*dx*(mm)	*dy*(mm)	*θ*(°)	RMSE(mm)	*dx*(mm)	*dy*(mm)	*θ*(°)	RMSE(mm)

2	0	0	1.7	0.2	−0.3	0.7	1.8	−0.0	−0.1	0.3
4	0	0	3.9	0.2	0.2	0.4	4.7	−0.1	−0.1	0.7
6	0	0	6.4	−0.1	0.0	0.4	6.0	−0.0	−0.2	0.4
8	0	0	8.5	0.5	0.2	0.8	8.1	−0.2	0.1	0.3
10	0	0	10.5	0.3	−0.2	0.7	10.5	−0.2	−0.2	0.7
12	0	0	12.6	−0.2	−0.1	0.6	11.9	0.0	−0.1	0.2
0	2	0	−0.3	2.4	0.4	0.9	0.1	2.4	0.1	0.5
0	4	0	−0.1	4.6	0.3	0.8	0.2	4.5	0.2	0.7
0	6	0	0.1	6.8	0.1	0.8	0.6	6.8	0.2	1.0
0	8	0	0.6	8.5	0.25	0.9	0.2	8.4	−0.1	0.5
0	10	0	0.0	11.3	−0.1	1.4	0.2	10.9	0.1	0.9
0	12	0	0.8	12.6	0.4	1.3	0.5	12.8	0.2	0.9

**Table 3:** Setup error estimations (horizontal displacement, vertical displacement, angle of rotation, and RMS error) for six subjects. The values listed are the mean values ± standard deviation calculated over a set of thirty portal images. The RMSEmeasurements for portal-portal versus DRR-portal did not show significant differences (*P* > 0.05,Wilcoxon signed test).

Patient	Setup error	*dx*(mm)	*dy*(mm)	*θ*(°)	RMSE(mm)

1	Expected	−1.8 ± 0.2	−1.6 ± 0.1	−0.1 ± .0.2	
	DRR versus portal	−1.8 ± 0.12	−1.5 ± 0.1	−0.2 ± .0.2	0.1 ± 0.1
	Portal versus portal	−1.6 ± 0.2	−1.8 ± 0.1	0.2 ± .0.2	0.3 ± 0.1
2	Expected	2.0 ± 0.3	−1.0 ± 0.5	−0.2 ± 0.1	
	DRR versus portal	2.0 ± .0.4	−1.2 ± .0.6	−0.2 ± .0.2	0.1 ± 0.1
	Portal versus portal	1.9 ± .0.2	−1.1 ± .0.3	−0.2 ± .0.1	0.1 ± 0.1
3	Expected	−1.9 ± 0.5	−0.1 ± 0.0	−0.1 ± .0.2	
	DRR versus portal	−2.0 ± .0.4	−0.1 ± .0.1	−0.1 ± .0.1	0.8 ± 0.5
	Portal versus portal	−2.0 ± .0.4	−0.1 ± .0.1	−0.1 ± .0.2	0.7 ± 0.5
4	Expected	−0.1 ± .0.1	−3.5 ± 0.7	−0.2 ± .0.3	
	DRR versus portal	−0.1 ± .0.1	−3.5 ± .0.6	−0.2 ± .0.2	0.1 ± 0.0
	Portal versus portal	−0.1 ± .0.1	−3.8 ± .0.6	−0.2 ± .0.2	0.2 ± 0.0
5	Expected	0.5 ± .0.1	1.6 ± 0.6	0.4 ± .0.3	
	DRR versus portal	0.8 ± .0.1	1.9 ± .0.7	0.4 ± .0.3	0.6 ± 0.1
	Portal versus portal	0.7 ± .0.1	1.7 ± .0.7	0.4 ± .0.2	0.3 ± 0.1
6	Expected	2.0 ± .0.3	−1.5 ± 0.6	0.2 ± .0.2	
	DRR versus portal	2.4 ± .0.4	−1.1 ± .0.5	0.2 ± .0.2	0.3 ± 0.1
	Portal versus portal	2.1 ± .0.2	−1.4 ± .0.4	0.1 ± .0.1	0.3 ± 0.0	

## References

[1] Herman MG (2005). Clinical use of electronic portal imaging. *Seminars in Radiation Oncology*.

[2] Hurkmans CW, Remeijer P, Lebesque JV, Mijnheer BJ (2001). Set-up verification using portal imaging; review of current clinical practice. *Radiotherapy and Oncology*.

[3] Hanley J, Mageras GS, Sun J, Kutcher GJ (1995). The effects of out-of-plane rotations on two dimensional portal image registration in conformal radiotherapy of the prostate. *International Journal of Radiation Oncology, Biology, and Physics*.

[4] Fritsch DS, Chaney EL, Boxwala A (1995). Core-based portal image registration for automatic radiotherapy treatment verification. *International Journal of Radiation Oncology, Biology, and Physics*.

[5] Sirois LM, Hristov DH, Fallone BG (1999). Three-dimensional anatomy setup verification by correlation of orthogonal portal images and digitally reconstructed radiographs. *Medical Physics*.

[6] Lemnitzer H, Wolf U, Hildebrandt G, Kamprad F (1999). Verification of electron field positioning. *Radiotherapy and Oncology*.

[7] Dong L, Boyer AL (1995). An image correlation procedure for digitally reconstructed radiographs and electronic portal images. *International Journal of Radiation Oncology, Biology, and Physics*.

[8] Wang J-Z, Reinstein LE, Hanley J, Meek AG (1996). Investigation of a phase-only correlation technique for anatomical alignment of portal images in radiation therapy. *Physics in Medicine and Biology*.

[9] Bansal R, Staib LH, Chen Z A novel approach for the registration of 2D portal and 3D CT images for treatment setup verification in radiotherapy.

[10] Clippe S, Sarrut D, Malet C, Miguet S, Ginestet C, Carrie C (2003). Patient setup error measurement using 3D intensity-based image registration techniques. *International Journal of Radiation Oncology, Biology, and Physics*.

[11] Khamene A, Bloch P, Wein W, Svatos M, Sauer F (2006). Automatic registration of portal images and volumetric CT for patient positioning in radiation therapy. *Medical Image Analysis*.

[12] Lujan AE, Balter JM, Ten Haken RK (1998). Determination of rotations in three dimensions using two-dimensional portal image registration. *Medical Physics*.

[13] Remeijer P, Geerlof E, Ploeger L, Gilhuijs K, Van Herk M, Lebesque JV (2000). 3-D portal image analysis in clinical practice: an evaluation of 2-D and 3-D analysis techniques as applied to 30 prostate cancer patients. *International Journal of Radiation Oncology, Biology, and Physics*.

[14] Matsopoulos GK, Asvestas PA, Delibasis KK (2004). Registration of electronic portal images for patient set-up verification. *Physics in Medicine and Biology*.

[15] Gonzalez RC, Woods RE (2002). *Digital Image Processing*.

[16] Pizer SM, Amburn EP, Austin JD (1987). Adaptive histogram equalization and its variations. *Computer Vision, Graphics, and Image Processing*.

[17] Canny J (1986). A computational approach to edge detection. *IEEE Transactions on Pattern Analysis and Machine Intelligence*.

[18] van Vliet LJ, Young IT, Verbeek PW Recursive Gaussian derivative filters.

[19] Borgefors G (1984). Distance transformations in arbitrary dimensions. *Computer Vision, Graphics, & Image Processing*.

[20] Press WH, Teukolsky SA, Vetterling WT, Flannery BP (1993). Minimization or maximization of functions. *Numerical Recipes in C: The Art of Scientific Computing*.

[21] Matsopoulos GK, Asvestas PA, Mouravliansky NA, Delibasis KK (2004). Multimodal registration of retinal images using self organizing maps. *IEEE Transactions on Medical Imaging*.

[22] Kohonen T (2000). *Self-Organizing Maps*.

[23] Ingber L, Rosen B (1992). Genetic algorithms and very fast simulated reannealing: a comparison. *Mathematical Computer Modelling*.

[24] Siegel S, Castellan NJ (1988). *Nonparametric Statistics for the Behavioral Science*.

[25] Dong L, Boyer AL (1995). An image correlation procedure for digitally reconstructed radiographs and electronic portal images. *International Journal of Radiation Oncology, Biology, and Physics*.

[26] Fritsch DS, Raghavan S, Boxwala A (1997). Benchmark test cases for evaluation of computer-based methods for detection of setup errors: realistic digitally reconstructed electronic portal images with known setup errors. *International Journal of Radiation Oncology, Biology, and Physics*.

[27] Clippe S, Sarrut D, Malet C, Miguet S, Ginestet C, Carrie C (2003). Patient setup error measurement using 3D intensity-based image registration techniques. *International Journal of Radiation Oncology, Biology, and Physics*.

